# Foliar Application of Salicylic Acid Improves Water Stress Tolerance in *Conocarpus erectus* L. and *Populus deltoides* L. Saplings: Evidence from Morphological, Physiological, and Biochemical Changes

**DOI:** 10.3390/plants10061242

**Published:** 2021-06-18

**Authors:** Zikria Zafar, Fahad Rasheed, Rana Muhammad Atif, Muhammad Asif Javed, Muhammad Maqsood, Oliver Gailing

**Affiliations:** 1 Department of Forestry & Range Management, University of Agriculture, Faisalabad 38040, Pakistan; z.zafarfrw@gmail.com (Z.Z.); azurefromheavens@gmail.com (M.A.J.); 2 Department of Forest Genetics and Forest Tree Breeding, University of Göttingen, Büsgenweg, 2 D-37077 Göttingen, Germany; 3 Department of Plant Breeding and Genetics, University of Agriculture, Faisalabad 38040, Pakistan; dratif@uaf.edu.pk; 4 Center for Advanced Studies in Agriculture and Food Security (CAS-AFS), University of Agriculture, Faisalabad 38040, Pakistan; 5 Department of Agronomy, University of Agriculture, Faisalabad 38040, Pakistan; cool_kantay@hotmail.com

**Keywords:** drought stress, osmolytes, oxidants, antioxidants, poplar, leaf gas exchange

## Abstract

Reforestation efforts are being challenged as water stress is hampering the sapling growth and survival in arid to semiarid regions. A controlled experiment was conducted to evaluate the effect of foliar application of salicylic acid (SA) on water stress tolerance of *Conocarpus erectus* and *Populus deltoides*. Saplings were watered at 90%, 60%, and 30% of field capacity (FC), and half of the saplings under 60% and 30% FC were sprayed with 1.0 mM SA. Results indicated that dry weight production decreased significantly in *Populus deltoides* under both water deficit conditions, and leaf gas exchange parameters decreased significantly in both the species under both soil water deficit conditions. Foliar application of SA resulted in a significant increase in leaf gas exchange parameters, and compatible solutes, thereby increasing the dry weight production in both of the species under soil water deficit. Oxidative stress (hydrogen peroxide and superoxide anions) increased under soil water deficit and decreased after the foliar application of SA and was parallel to the increased antioxidant enzymes activity (superoxide dismutase, catalase, peroxidase, and ascorbate peroxidase). Therefore, it can be concluded that foliar application of 1.0 mM SA can significantly improve the water stress tolerance in both species, however, positive impacts of SA application were higher in *Conocarpus erectus* due to improved photosynthetic capacity and increased antioxidant enzyme activity.

## 1. Introduction

Accentuation of climatic extremes has resulted in a global decrease in water availability and increase in evaporative demand [1]. Moreover, warmer climate and fluctuating summer rainfall have resulted in a global increase in water scarcity [2]. South Asia is ranked 5 th among the severely affected regions of the world due to climate change [3]. According to various predictive models, rise in the drought frequency and severity is expected during the coming years, making drought one of the most important abiotic stress factors limiting plant growth and development [4,5,6]. To mitigate the impacts of climate change, Pakistan has started an ambitious tree-planting campaign all across the country (Ten Billion-tree Tsunami Project; https://en.unesco.org/courier/2019-3/pakistan-green-again, accessed on 20 May 2021). During this project, almost one billion trees have already been planted across the country. However, Pakistan falls under arid and semiarid climates, therefore reduced growth and mortality of newly planted saplings due to water stress has become a major problem. This calls for an urgent need to explore methods of improving the water stress tolerance of tree saplings that can increase the chances of their survival and growth under stressful environments. 

Globally, tree species are growing within narrow hydraulic limits and are susceptible to long-term productivity loss under climate change [7,8,9]. Adverse effects of climate change such as hydraulic failure [10], altered productivity and increased tree mortality [11] have already been reported. Plants generally respond to abiotic stresses by adjusting morphological, physiological, and biochemical attributes [12,13,14]. In this regard, reduction in stomatal conductance allows the species to regulate water loss, however, such adjustments may result in CO_2_ starvation, decrease in plant growth, biomass production, total leaf area, and increase in root:shoot ratio under severe conditions [15,16,17,18,19]. Increased production of various osmolytes like proline, carbohydrates, and soluble sugar has been linked with retaining water in the cytoplasm and preventing protein denaturation and cell membrane damage [20,21,22]. Increased production of reactive oxygen species (ROS) like hydrogen peroxide (H_2_O_2_), superoxide radical (O_2_^−^), singlet oxygen (_1_O^2^), and hydroxyl radicals (OH), and increased production of various antioxidant enzymes like superoxide dismutase (SOD), catalase (CAT), and peroxidases (POD), and ascorbate peroxidase (APX) has also been evidenced under water stress to prevent cellular damage [23,24,25]. Therefore, to overcome the drought-induced adversities and to reduce sapling mortality due to water stress, enhancing drought tolerance in plants becomes a prerequisite [26]. In this regard, various approaches, including exogenous use of plant growth regulators, have shown promising results in mitigating the detrimental effects of drought stress on different plant species. 

Salicylic acid (SA) is an important phytohormone that has received much attention among the stress-mitigating hormones in the past decades. Being a vital signaling molecule, fluctuation in the production of SA affects various physiological processes such as photosynthesis, accumulation of antioxidant enzymes, proline metabolism, thereby sustaining plant growth and development under abiotic stresses such as drought and salinity [15,27,28]. Studies have demonstrated that SA supplementation also enhances the photosynthetic activity, chlorophyll content, leaf water potential, membrane stability index, activities of antioxidant enzymes like SOD, POD, CAT, and APX, and reduces the electrolyte leakage and lipid peroxidation under water stress conditions [28,29,30]. Therefore, the application of SA has been considered a short-term solution to reduce the negative effects of water stress in plants [15,30,31], however, the accurate mode of SA action remains unclear, particularly in tree species [32,33]. In this scenario, the current study explored the water stress tolerance of *Conocarpus erectus* and *Populus deltoides* saplings after the foliar application of salicylic acid. These two species are being widely used in the tree planting campaign and therefore were selected for this study. *Conocarpus erectus* is an evergreen shrub/tree that belongs to the family *Combretaceae*. The species is native to Northern Somalia and widely distributed over the subtropical and arid to semiarid regions of the world [34]. Leaves are shiny on both sides due to the formation of trichomes and root formation is profuse and fibrous in nature [35]. *Conocarpus* has the ability to grow in compacted soils and can also tolerate drought [36], therefore, the species is used for the reforestation of arid to semiarid areas. *Populus deltoides* belongs to the family *Salicaceae*. The species is native to North America but is widely distributed all over the world. *Populus deltoides* is known for its high growth potential which is dependent on water availability [17]. Globally, *Populus* genotypes are widely being tested and selected for better stress tolerance against various abiotic factors [37,38]. In this study, plant saplings were subjected to water stress and the effect of foliar application of 1 mM of SA was evidenced on various physiological, morphological, and biochemical traits to evaluate the tolerance status of these two tree species under controlled conditions. Although the experiment was conducted under controlled conditions, the results will serve as perquisite for the future research under field conditions implicated in evidencing species’ tolerance to soil water deficit and effectivity of SA application. 

## 2. Materials and Methods

### 2.1. Growth Conditions 

A pot experiment was conducted in a greenhouse during August–September 2018 at Forestry farm, Department of Forestry and Range Management (31°26′ N, 73°06′), University of Agriculture Faisalabad, Pakistan. Mean temperature, relative humidity, photosynthetic photon flux density (PPFD) remained at 25 ± 5 °C, 65 ± 5% and 1200 PPFD, respectively. Seventy pots (25 × 30 cm; weight 260 ± 5 g) were filled with 8 kg mixture of sandy loam soil and farmyard manure (3:1). The soil mixture was also tested for nitrogen 0.78%, phosphorus (12 ppm), organic matter (15.5%), electric conductivity 2 dSm^−1^, and pH (6.6).

### 2.2. Water Stress and Salicylic Acid Application 

Water stress in all the pots was maintained at three levels: control (90% of field capacity), MS (60% of field capacity), and HS (30% of field capacity) following the methods of Rasheed et al. (37). Each pot was watered back daily to the reference weight by weighing the pots and adding the amount of water lost during evapotranspiration. A total of 90 saplings of *Cononcarpus erectus* and *Populus deltoides* were procured from the Punjab Forestry Research Institute Gatwala, Faisalabad, and 70 plants (35 plants per species) were selected (plant height, 35 ± 5 cm) for this experiment. Seven plants per species were randomly assigned either to control (C; 90% field capacity), medium water stress (MS, 60% field capacity), high water stress (HS; 30% field capacity), medium water stress + 1.0 mM SA (MS + SA), and high water stress + 1.0 mM SA (HS + SA) treatments. Before the experiment, 5 g of NPK fertilizer (15% N, 5% P_2_O_5_, 5% K_2_O) was also added to each pot. Sodium salicylate (Merck, Darmstadt, Germany) was used to prepare the salicylic acid solution (SA) by dissolving 138.1 mg of salicylic acid in 1 L of distilled water. The pH of the SA was adjusted to 7.0 by adding NaOH solution. On the 7 th and 45 th day of the experiment, the plants under control, MS and HS, were sprayed with distilled water, and the plants under MS + SA and HS + SA treatment were sprayed with 1.0 mM SA solution. The experiment continued for 90 days using a completely randomized design (CRD).

### 2.3. Growth and Biomass Production

Various growth traits like height (*H*, cm) and stem diameter (*D*, mm) were measured during the experiment. At the end of the experiment, each plant was harvested and separated into the different plant sections leaves, stem, and roots. All the plant sections were separately placed in paper bags and dried at 70 °C for 72 h in a heat oven (DGH-9202 Series Thermal Electric Thermostat drying oven). Subsequently, the dry weight of each plant section, leaf (D_L_) stem (D_S_), roots (D_R_), total dry weight (D_T_), and root/shoot ratio (R:S ratio), was calculated following Zafar et al. [39].

### 2.4. Leaf Gas Exchange Measurements 

Gas exchange measurements were conducted one week before the end of the experiment. A fully mature leaf was selected per plant and various parameters like net CO_2_ assimilation rate (*Ar*, μmol CO_2_ m^−2^ s^−1^) and stomatal conductance (g_s_, mol m^−2^ s^−1^) were measured using a portable Infrared Gas Analyzer (CIRAS-3 Amesbury, USA). All measurements were taken between 12:00 am and 01:00 pm. The leaf chamber temperature was set at 27 °C, relative humidity was kept at 65%, and the reference CO_2_ was adjusted at 400 μmol mol^−1^. Intrinsic water use efficiency (WUE_i_, μmol mol^−1^) was calculated as a ratio of net CO_2_ assimilation rate and stomatal conductance by Rasheed et al. [37]. 

### 2.5. Determination of Chlorophyll *a*, *b*, Carotenoid, and Various Osmolytes

At the end of the experiment, healthy and mature leaves were collected from each sapling, and chlorophyll *a* and *b* (*Chl a* and *b*), and carotenoid content (Cc) were determined following the method of Arnon [40]. Absorption was measured at 470, 645, and 663 nm using a spectrophotometer (Eppendorf BioSpectrometer ^®^ Basic; Hamburg, Germany). Proline content (Pc) in leaves was quantified using the ninhydrin method of Bates [41]. The reaction mixture was extracted with 5 mL toluene, cooled at room temperature and absorbance was measured at 520 nm using a spectrophotometer (Eppendorf BioSpectrometer ^®^ Basic; Hamburg, Germany) and total phenolic contents (TPC) were determined using the Folin–Ciocalteu reagent method of Ainsworth and Gillespie [42]. Total soluble proteins (SP) were determined by following the method of Bradford [43] and total soluble sugars (TSS) were determined using the Anthrone method of Yemm and Willis [44]. 

### 2.6. Determination of Malondialdehyde Contents and Electrolyte Leakage 

Lipid peroxidation was determined in terms of malondialdehyde contents (MDA) following the method of Hodges [45]. Electrolyte leakage (EL%) was determined by taking 200 mg of fresh leaf sample that was placed in 20 mL of deionized water and the tubes were placed in a water bath at 32 °C. After two hours, the initial electrical conductivity (EC_i_) was measured. Final electrical conductivity (EC_f_) was measured after placing the samples in a heated oven at 100 °C for twenty minutes. EL % was calculated using the following equation by Nayyar [46].

EL (%) = (EC_i_/EC_f_) × 100

### 2.7. Production of Reactive Oxygen Species (ROS)

The production of hydrogen peroxide (H_2_O_2_) was determined as described by Velikova [47]. In total, 5 mL of 0.1% TCA (Trichloroacetic acid) was added to 200 mg of leaf sample and placed in an ice bath. Samples were then centrifuged at 12,000× *g* for 15 min and 1 mL of 10 mM potassium phosphate buffer (pH 7.0) and 2 mL of 1 M potassium iodide was added to 1 mL of the supernatant. Samples were placed in a dark room for one hour and the absorbance of the supernatant was measured at 390 nm using a spectrophotometer (Eppendorf BioSpectrometer ^®^ Basic; Hamburg, Germany). Superoxide radicals (O_2_^−^) in leaf samples were determined according to Bai [48]. In total, 1.0 g of leaf samples was homogenized in 4 mL of 65 mM phosphate buffer and centrifuged at 5000× *g* for 15 min. After centrifugation we obtained 1 mL of supernatant and added 0.1 mL of 10 mM hydroxylamine chlorine and 0.9 mL of 65 mM phosphate buffer (pH 7.8) and kept the mixture for 20 min at 25 °C in a water bath. Then, 1.0 mL of 17 mM sulfanilic acid and 1.0 mL of 7 mM α-naphthylamine were added to 1.0 mL of the solution and held for 20 min at 25 °C. The absorbance was determined at 530 nm.

### 2.8. Antioxidant Enzyme Activity

The activity of superoxide dismutase (SOD) was measured by photochemical reduction of NBT (nitroblue tetrazolium) as described by Bayer [49]. One unit of SOD was measured via the 50% photochemical reduction of nitroblue tetrazolium (NBT) at 560 nm (Eppendorf BioSpectrometer^®^ Basic; Hamburg, Germany). Peroxidase (POD) activity was measured as described by Maehly and Chance [50]. The enzyme activity was determined by the increase in absorption at 470 nm for 1 min by using a spectrophotometer (Eppendorf BioSpectrometer ^®^ Basic; Hamburg, Germany). Catalase (CAT) activity was determined following the method of Knörzer [51]. The 0.5 g leaf samples were mixed with phosphate buffer (2.85 mL of 100 mM) and the mixture was centrifuged at 10,000 rpm for 10 min at 4 °C and supernatant was obtained. The reaction mixture was prepared with 1.5 mL 1000 mM of phosphate buffer (pH 7.8), 200 μL of H_2_O_2_ and 200 μL enzyme extract. The CAT activity was determined through the quantity of H_2_O_2_ consumed at 240 nm per min. Ascorbate peroxidase (APX) activity was determined according to the method of Nakano and Asada [52].

### 2.9. Statistical Analysis

Normality was checked using Q-Q plots and the data were analyzed using a two-way ANOVA for species, treatment, and interaction effect. The differences between means were compared using the post-hoc *Tukey’s* HSD test. All means are presented with their standard errors and tests were considered as significant at *p* < 0.05. All tests were run using Statistica 12.5 (Statsoft, Maisons-Alfort, France).

## 3. Results

### 3.1. Effect of Soil Water Deficit and SA Application on Growth and Dry Weight Production

The results showed that mean *H* (plant height) and *D* (stem diameter) varied significantly among the species. In *Conocarpus erectus*, mean *H* and *D* decreased significantly under HS as compared to control and increased under HS + SA (20.7% and 21.3%) as compared to HS. In *Populus deltoides*, mean plant height, *H* and stem diameter, *D* decreased significantly in both MS and HS as compared to the control and increased significantly only under HS + SA (34.9% and 16.2%) as compared to HS (Table 1). 

**Figure 1:**
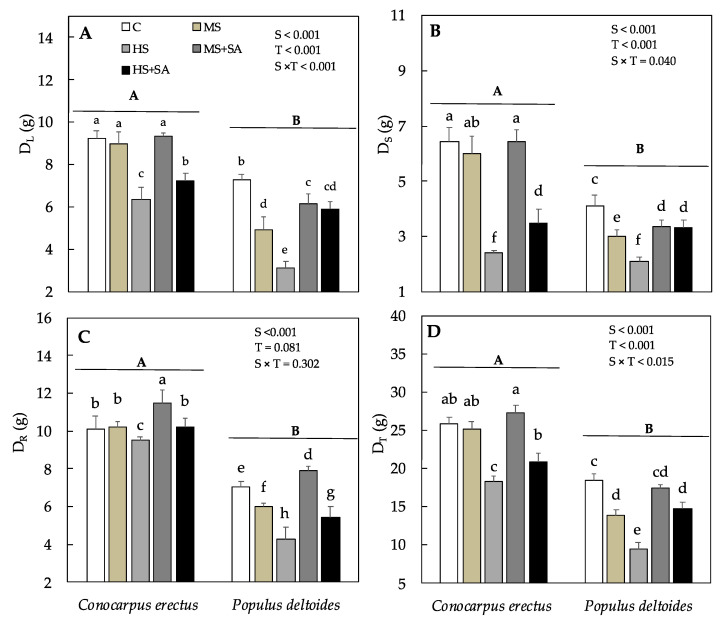
Effect of soil water deficit and SA application on (**A**) mean leaf dry weight, D_L_, (**B**) mean stem dry weight, D_S_, (**C**) mean root dry weight, D_R_, and (**D**) mean total dry weight, D_T_ in *Conocarpus erectus* and *Populus deltoides*, respectively. Each value represents the mean (± SE) of species in different treatment combinations. Capital and small letters set in bold represent significant differences among the species and treatments tested using the post-hoc Turkey’s HSD test. All tests were considered significant at *p* < 0.05.

Mean dry weight production in leaf (D_L_), stem (D_S_), root (D_R_), and total dry weight (D_T_) differed significantly among species and treatments. In *Conocarpus erectus*, D_L_, D_S_, D_R_, and D_T_ decreased significantly in plants under HS as compared to the control and increased significantly under HS + SA (13.8%, 44.3%, 6.91% and 14.8%) as compared to HS (Figure 1A–D). However, in *Populus deltoides*, a significant decrease in D_L_, D_S_, D_R_, and D_T_ was observed in both MS and HS as compared to the control and a significant increase was observed in both MS + SA (24.8%, 13.0%, 31.6% and 25.1%) and HS + SA (90.3%, 57.5%, 26.6% and 53.3%) as compared to MS and HS, respectively (Figure 1A–D). Resultantly, in *Conocarpus erectus* root:shoot ratio (R:S ratio) increased significantly under both MS (6.25%) and HS (70.3%) as compared to the control, however, remained similar under MS + SA and HS + SA as compared to MS and HS, respectively (Table 1). In *Populus deltoides*, R:S ratio increased significantly in both MS (22.9%) and HS (32.7%) as compared to the control. However, a significant increase was observed under MS + SA (9.33%) as compared to MS whereas a significant decrease was observed under HS + SA (28%) as compared to HS (Table 1).

### 3.2. Effect of Soil Water Deficit and SA Application on Chlorophyll *a*, *b*, and Carotenoids Contents

Mean chlorophyll *a* and *b* (*Chl a* and *b*) along with carotenoid contents (Cc) varied significantly among species as well as across treatments (Table 1). In *Conocarpus erectus*, mean *Chl a*, *b* and Cc decreased significantly in plants under MS and HS as compared to the control whereas a significant increase was observed in plants under MS + SA (12.6%, 5.29% and 15.5%) and HS + SA (23.1%,18.7% and 30.7%) as compared to MS and HS, respectively (Table 1). Similar results were observed in *Populus deltoides* where mean *Chl a*, *b* and Cc decreased significantly under both MS and HS as compared to the control and increased significantly in MS + SA (17.5%, 18.3% and 25.2%) and HS + SA (40.6%, 44.0% and 27.2%) as compared to MS and HS, respectively (Table 1).

### 3.3. Effect of Soil Water Deficit and SA Application on the Production of Osmolytes 

Mean proline contents (Pc), total soluble sugars (TSS), total phenolic contents (TPC), and soluble proteins (SP) also varied significantly between the species. In *Conocarpus erectus*, mean concentration of Pc, TSS, TPC, and SP increased progressively in plants under MS and HS as compared to the control, and a significant increase was evidenced in Pc, TSS and TPC under MS + SA (22.2%, 4.69% and 15.4%) and HS + SA (10.0%, 5.10% and 10.5%) as compared to MS and HS, respectively (Table 2). Similarly, in *Populus deltoides*, mean concentrations of Pc, TSS, TPC, and SP increased progressively under MS and HS as compared to the control, and further increased significantly under MS + SA (31.6%, 5.22%, 15.0% and 2.97%) and HS + SA (13.5%, 6.56%, 11.9%, and 2.56%) as compared to MS and HS, respectively (Table 2).

### 3.4. Effect of Soil Water Deficit and SA Application on the Variations in Leaf Gas Exchange Parameters

**Figure 2:**
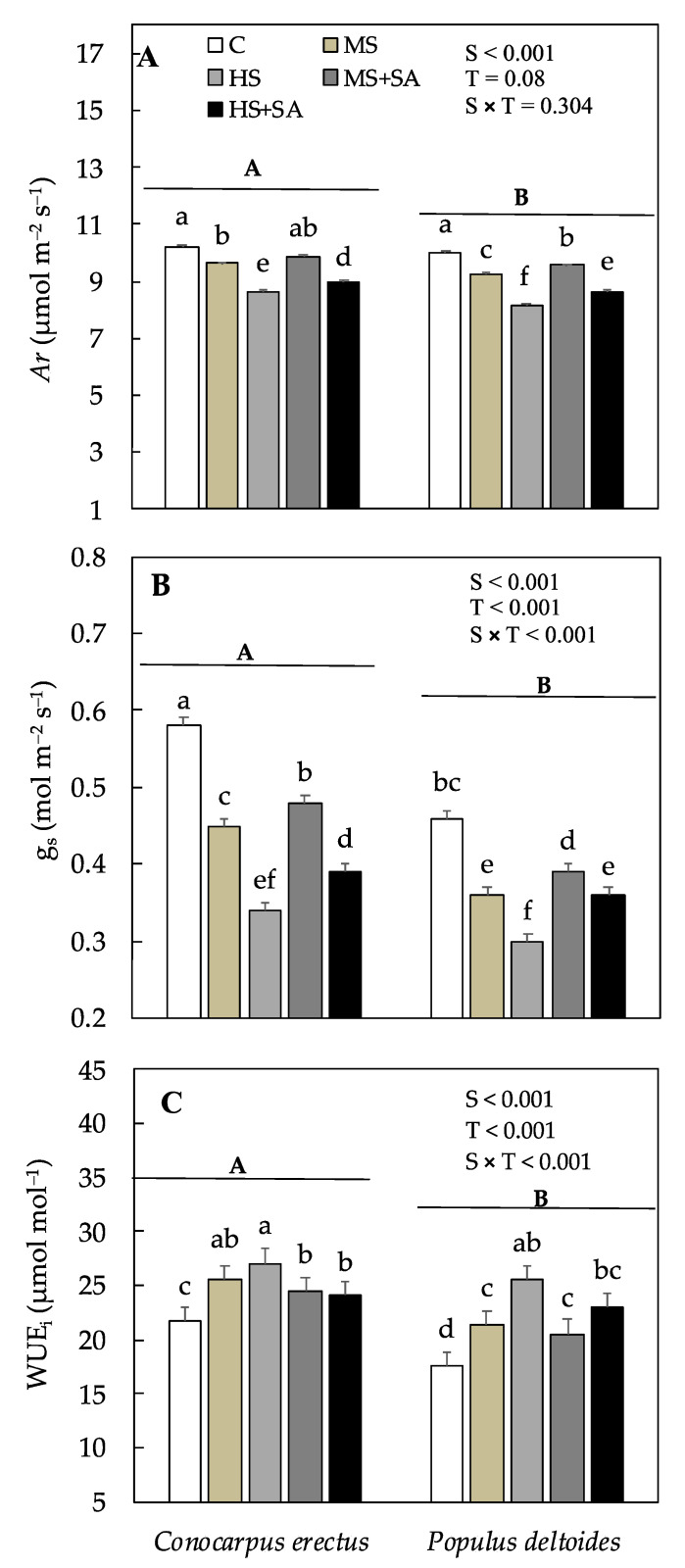
Effect of water deficit and SA application on gas exchange parameters (**A**) CO_2_ assimilation rate, *Ar*, (**B**) stomatal conductance, g_s_, and (**C**) intrinsic water use efficiency, WUE_i_ in *Conocarpus erectus* and *Populus deltoides*, respectively. Each value represents the mean (± SE) of species in different treatment combinations. Capital and small letters set in bold represent significant differences among the species and treatments tested using the post-hoc Turkey’s HSD test. All tests were considered significant at *p* < 0.05.

Net CO_2_ assimilation rate (*Ar*) remained similar in both species and varied significantly across the treatments, however, stomatal conductance (g_s_) varied significantly across the species as well as treatments. In *Conocarpus erectus*, mean *Ar* and g_s_ decreased significantly in plants under MS and HS as compared to the control whereas a significant increase was observed under MS + SA (2.38% and 6.66%) and HS + SA (3.81% and 14.7%) as compared to MS and HS, respectively (Figure 2A,B). In *Populus deltoides*, mean *Ar* and g_s_ decreased significantly in plants under MS and under HS as compared to the control, and a significant increase was observed under MS + SA (3.68% and 8.33%) and HS + SA (6.65% and 20%) as compared to MS and HS, respectively (Figure 2A,B). Resultantly, intrinsic water use efficiency (WUE_i_) varied significantly between the species as well as across the treatments (Table 1). In both *Conocarpus erectus* and *Populus deltoides*, WUE_i_ progressively increased under HS as compared to the control and decreased significantly by 11.1% and 9.44% under HS + SA as compared to HS, respectively (Figure 2C).

### 3.5. Effect of Soil Water Deficit and SA Application on the Concentration of MDA, EL % and Oxidant and Antioxidant Enzyme Activity 

**Figure 3:**
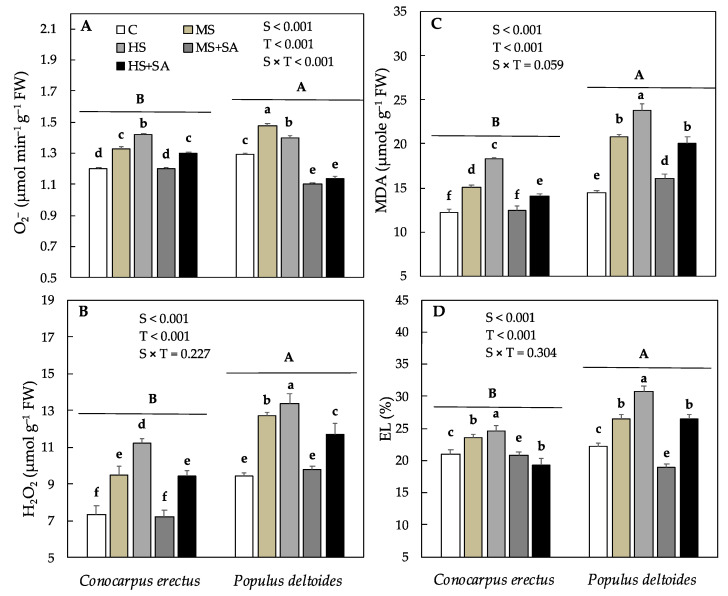
Effect of water deficit and SA application on (**A**) malondialdehyde contents, MDA, (**B**) electrolyte leakage, EL%, along with various oxidants, (**C**) superoxide radical, O_2_^−^ and (**D**) hydrogen peroxide, H_2_O_2_ in *Conocarpus erectus* and *Populus deltoides*, respectively. Each value represents the mean (± SE) of species in different treatment combinations. Capital and small letters set in bold represent significant differences among the species and treatments tested using the post-hoc Turkey’s HSD test. All tests were considered significant at *p* < 0.05.

The concentration of malondialdehyde (MDA), electrolyte leakage (EL%) and production of superoxide radical (O_2_^−^), and hydrogen peroxide (H_2_O_2_) varied significantly between the species as well as across the treatments. In *Conocarpus erectus*, means of MDA, EL%, O_2_^−^, and H_2_O_2_ progressively increased in plants under MS and HS as compared to the control, and decreased significantly under MS + SA (16.8%, 11.4%, 9.0% and 24.0%) and HS + SA (22.5%, 21.2%, 8.4% and 17.8%) as compared to MS and HS, respectively (Figure 3A–D). In *Populus deltoides* means of MDA, EL%, O_2_^−^, and H_2_O_2_ also increased progressively in MS and HS as compared to the control, and decreased significantly under MS + SA (22.2%, 28.4%, 25% and 23%) and HS + SA (15.1%, 13.6%, 18.5% and 12%) as compared to MS and HS, respectively (Figure 3A–D).

**Figure 4:**
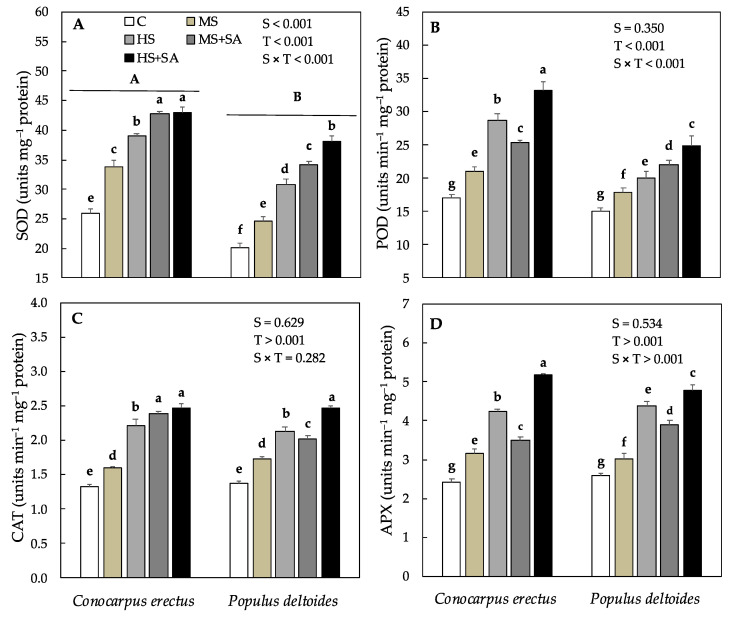
Effect of water deficit and SA application on various antioxidant enzyme activities. (**A**) Superoxide dismutase, SOD, (**B**) peroxidase, POD, (**C**) catalase, CAT, and (**D**) ascorbate peroxidase, APX in *Conocarpus erectus* and *Populus deltoides*, respectively. Each value represents the mean (± SE) of species in different treatment combinations. Capital and small letters set in bold represent significant differences among the species and treatments tested using the post-hoc Turkey’s HSD test. All tests were considered significant at *p* < 0.05.

The activity of superoxide dismutase (SOD), peroxidase (POD), catalase (CAT), and ascorbate peroxidase (APX) also varied significantly between the species as well as across the treatments. In *Conocarpus erectus*, the activity of SOD, POD, CAT, and APX progressively increased in plants under MS and HS as compared to the control, and increased further under MS + SA (27%, 20.9%, 49.3%, and 10.4%) and HS + SA (10.5%, 15.3%, 11.3%, and 21.9%) as compared to MS and HS, respectively (Figure 4A–D). In *Populus deltoides* activity of SOD, POD, CAT, and APX also increased progressively in MS and HS as compared to the control, and increased further under MS + SA (39%, 18.5%, 17.4%, and 28.7%) and HS + SA (23.3%, 24%, 15.4%, and 11.3%) as compared to MS and HS, respectively (Figure 4A–D).

## 4. Discussion

Morphological adjustments are the most common response in plants to water stress, however, such response depends on the intensity of stress and is often species-specific [53]. Our results showed that although *Conocarpus erectus* saplings showed tolerance to medium water stress, the total dry weight (D_T_) decreased by 29.1% in *Conocarpus erectus* and 48.3% in *Populus deltoides* under HS as compared to the control (Figure 1D). Similar negative effects of water stress have been reported previously in *Betula* spp. [18], *Ziziphus jujube* [14], *Eucalyptus globulus* [54], *Populus tremula*, *Betula pendula* [55], and *Syzygium cumini* [6]. Reduction in growth under water stress is often related to the decrease in water absorption from the soil, plant turgor pressure, and CO_2_ assimilation rate [12,13]. In this study, a significant decrease in root dry weight (D_R_) was observed in both *Conocarpus erectus* and *Populus deltoides* under HS (Figure 1C). Therefore, reduction in D_R_ may have resulted in reduced water uptake and plant turgor pressure under HS. Although D_R_ decreased significantly under water stress, the R:S ratio increased significantly in both *Conocarpus erectus* and *Populus deltoides* (Table 1). These results showed that both the species demonstrated a stress avoiding strategy in which plants prefer decreasing shoot growth in favor of root growth. Such a response has also been reported previously in other tree species like *Ziziphus* *jujube* [14]. Moreover, a significant decrease of 15.9% and 18.8% was also observed in the CO_2_ assimilation rate (*Ar*) in *Conocarpus erectus* and *Populus deltoides* under HS (Figure 2A). Excessive reduction in stomatal conductance (g_s_) has been accepted as a key limiting factor in sustaining photosynthesis and growth under water stress [56]. In *Conocarpus erectus* and *Populus deltoides*, the decrease in *Ar* was parallel to the decrease in g_s_ under HS (41.3% and 36.9%, respectively), which supports this hypothesis. Alongside, a significant decrease in chlorophyll *a* and *b* (*Chl a* and *b*) along with carotenoid (Cc) content was also observed under soil water deficit which means an overall decrease in the photosynthetic capacity of plants under water stress. Therefore, the observed decrease in *Ar* under water stress is related to the decrease in g_s_ and *Chl a*, *b*. These results are in line with previous studies where a decrease in chlorophyll content and stomatal conductance was found responsible for the decrease in growth and productivity in *Betula* spp. [18]. In this study, foliar application of SA significantly increased total dry weight (D_T_) in both species under MS + SA and HS + SA (Figure 1D). These results are in agreement with previous studies on *Rosmarinus officinalis* L., *Oryza sativa* L., *Olea europaea* L., and *Eucalyptus globulus* where the application of SA resulted in an increase in biomass production under water stress [22,28,30,57,58]. Previously, improvements in growth after SA application have been related to the increased plant water potential and cell division in the meristematic parts of the plant [30,59]. In this study, a significant increase in root (D_R_) and leaf dry weight (D_L_) was evidenced in both species under MS + SA and HS + SA which supports this hypothesis (Figure 2B). These results are also in agreement with previous studies where the increase in root growth has been linked to the increased capability of plants to uptake water under water stress [14]. Furthermore, a significant increase in g_s_ (14.7% and 20%) and *Ar* (3.81% and 6.65%) was also evidenced in *Conocarpus erectus* and *Populus deltoides* under MS + SA and HS + SA, respectively. It has been demonstrated in previous studies that the application of SA reduces chlorophyll degradation by inducing the accumulation of photosynthetic pigments under water stress [60]. Therefore, the observed increase in *Chl a* and *b* in this study indicated that SA helped to alleviate the negative impacts of water stress and helped to increase *Ar* in both the species under MS + SA and HS + SA. Such an increase in chlorophyll content depicts an enhanced light use efficiency in plants after foliar application of SA [61]. Thus, the increase in g_s_ mediated by the enhanced root growth along with the increase in *Chl a* and *b* may have resulted in the observed increase in *Ar* and growth in plants under MS + SA and HS + SA. Based on the results it can be concluded that both species are susceptible to high soil water deficit, however, application of SA resulted in an increase in D_T_ which was mediated by the increase in root growth, chlorophyll content, and leaf gas exchange parameters.

In plants, proline content (Pc) and total soluble sugars (TSS) play an important role in the defense system or increasing water stress tolerance [29,62,63]. In the present study, the concentration of Pc and TSS increased significantly in both *Conocarpus erectus* and *Populus deltoides* under soil water deficit (Table 2). Among the two species, the concentration of Pc and TSS was higher in *Conocarpus erectus* as compared to *Populus deltoides*. The observed increase in Pc and TSS under water deficit is in line with previous studies on *L*. *citriodora*, mulberry, olive, and black poplars plants under soil water deficit [64,65,66,67]. Studies have shown that proline and soluble sugars act as osmotic molecules that play a significant role in increasing cell turgor and thus protecting the plants under water stress [62]. In this study, a significant increase in the concentration of Pc and TSS was observed in MS + SA and HS + SA as compared to MS and HS (Table 2). The increase in leaf soluble sugars under water stress is related to the breakdown of polysaccharides such as starch into glucose, which plays a significant role in increasing the osmotic potential and cellular turgor pressure under water stress [68]. Furthermore, a parallel increase in proline accumulation has also been observed previously after foliar application of SA under water stress [69]. Therefore, an increase in Pc and TSS under SA application may help to regulate the water balance reducing water stress-induced loss in productivity. Similar results have been reported in a previous study in which a significant increase in proline and soluble sugar contents after the application of SA has been linked to the growth sustainability in *Eucalyptus globulus* under water stress [22]. Phenolic compounds are important secondary metabolites that also play an important role in water stress tolerance in plants as they act as antioxidants and protect the plants against drought induced oxidative stress [33]. In this study, total phenolic content (TPC) progressively increased under MS and HS and further increased under MS + SA and HS + SA. These results are in agreement with previous studies in which an increase in phenolic contents has been reported after the application of SA [70]. Such increase in phenolic compounds under SA application has been linked to the upregulation of phenolic synthesizing enzymes under stress conditions [71]. Since phenolic compounds may act as antioxidants, increase in phenolic compounds under SA application can protect the plants under water stress-induced oxidative stress. In this study, this argument was supported by the observed increase in TPC under water stress concurrent with the decrease in MDA under MS + SA and HS + SA. Similar results are reported in previous studies where SA application significantly decreased the concentration of MDA indicating reduced membrane damage [22]. Based on these results, it can be concluded that the application of SA resulted in an increased production of various osmolytes (Pc, TSS, and TPC) that may have helped in improving the cellular osmotic status and productivity of both species under MS + SA and HS + SA.

The relationship between oxidants and antioxidants plays an important role in the tolerance status of species to abiotic stresses. Studies have demonstrated that water stress induces the production of reactive oxygen species (ROS) such as superoxide radical (O_2_^−^), hydrogen peroxide (H_2_O_2_) that damage proteins, the cell membrane, and lipids [19]. In the present study, the production of O_2_^−^ and H_2_O_2_ increased significantly in both species under HS (Figure 3). These results are in line with a previous study on six woody plant species where a similar increase in ROS has been evidenced under drought stress [24]. Other studies have demonstrated that increased production of ROS disrupts the redox balance and results in loss of productivity under water stress [6,19,72]. In this study, the significant increase in ROS and loss in dry weight production in *Conocarpus erectus* and *Populus deltoides* under HS supports this hypothesis. Moreover, studies have shown that the production of ROS causes peroxidation of lipid membranes by stimulating the decomposition of unsaturated lipids thus increasing the concentration of MDA and EL% (indicator traits for determining membrane stability) under water stress [73,74]. In the present study, the observed increase in MDA and EL % showed the ROS-assisted lipid peroxidation of membrane lipids in *Conocarpus erectus* and *Populus deltoides* under HS. Many previous studies have reported a similar increase in MDA and EL % in plants subjected to water stress [6,19,24,64]. However, under soil water deficit, the concentration of MDA and EL % was found higher in *Populus deltoides* as compared to *Conocarpus erectus* which depicts a greater vulnerability of this species to water stress. It has been shown previously that depending on the intensity of stress, an increase in the production of ROS may result in an increase or decrease in the activity of antioxidant enzymes that scavenge ROS and prevent cellular damage in plants under water stress [6,19,25]. In this study, parallel to the increase in the production of oxidants, antioxidant enzyme activity also increased significantly in *Conocarpus erectus* and *Populus deltoides* under water stress (Figure 4). Previous studies have linked the concurrent increase in the production of oxidants and antioxidant enzyme activity to an effective antioxidant mechanism under given stress conditions [19]. Thus, the concurrent increase in the activity of antioxidant enzymes under both MS and HS suggested that the detoxification mechanisms in both species remained effective even under HS.

In this study, foliar application of SA significantly reduced the concentration of H_2_O_2_ and O_2_^−^ in both species (Figure 3). These results are in agreement with previous studies in which a significant reduction in the production of ROS has been reported after the foliar application of SA [28,67]. Previous studies have related the decrease in ROS production to the increase in stress tolerance in plant species [25,33]. In this study, the decrease in H_2_O_2_ and O_2_^−^ under MS + SA and HS + SA along with a significant increase in total dry weight production supports this hypothesis. Furthermore, in the current study, the activity of antioxidant enzymes also increased significantly under MS + SA and HS + SA as compared to MS and HS, respectively (Figure 4). Previous reports have suggested that the application of SA may increase the activity of antioxidant enzymes like SOD, POD, CAT, and APX which protect the plant against ROS generation and lipid peroxidation [29,32]. A significant decrease was observed in MDA and EL% in both the species under MS + SA and HS + SA which supports this hypothesis. The involvement of SA in improving antioxidant enzyme activity and reducing the ROS activities has also been reported previously in Sesame plants under water stress [75]. Furthermore, Dianat et al. [67] observed that the foliar application of SA boosted the antioxidant defense mechanism in *Lippia citriodora* against oxidative damage induced by water stress. Therefore, it can be concluded that foliar application of SA significantly decreased the production of H_2_O_2_, O_2_^−^, MDA, and EL% which was mediated by the increased production of antioxidants in both species under water stress.

## 5. Conclusions

Results showed that soil water deficit had a significant negative effect on the growth and dry weight production in *Conocarpus erectus* and *Populus deltoides* saplings, however, the foliar application of SA improved the water stress tolerance of these two species. In both species, such improvement was partially mediated by a significant increase in R:S ratio, photosynthetic pigments (*Chl a*, *b* and carotenoids), and other osmolytes (proline and total soluble sugars and phenolic and proteins contents) under MS + SA and HS + SA that helped to increase stomatal conductance and CO_2_ assimilation rate. Furthermore, the application of SA significantly reduced the production of H_2_O_2_, O_2_^−^ and triggered the activity of antioxidant enzymes like SOD, POD, CAT, and APX in both *Conocarpus erectus* and *Populus deltoides*. Resultantly, in both *Conocarpus erectus* and *Populus deltoides* the concentration of MDA and EL % was found lowest in plants under MS + SA and HS + SA. Acquired data suggest that foliar application of SA can play an important role in enhancing the water stress tolerance in *Conocarpus erectus* and *Populus deltoides* saplings. However, the experiment was performed under controlled conditions, therefore, further experiments are required to validate the effectiveness of using the foliar application of SA under field conditions.

## Figures and Tables

**Table 1:** Effect of soil water deficit and SA application on various morphological and physiological parameters of *Conocarpus erectus* and *Populus deltoides*. Each value represents the mean ± SE of species in different treatments. Small letters represent significant differences among the species and treatments tested using the post-hoc Turkey’s HSD test. All tests were considered significant at *p* < 0.05.

Species	Treatments	*H* (cm)	*D* (mm)	R:S Ratio	Chl *a* (mg g^−^^1^ FW)	Chl *b* (mg g^−^^1^ FW)	Cc (mg g^−^^1^ FW)
	C	74.1 ± 2.33 a	6.18 ± 0.21 a	0.64 ± 0.07 d	2.18 ± 0.07 a	2.21 ± 0.02 a	0.82 ± 0.02 a
	MS	69.8 ± 2.05 ab	5.86 ± 0.40 ab	0.68 ± 0.05 c	1.66 ± 0.05 c	1.89 ± 0.04 b	0.72 ± 0.02 b
*Conocarpus*	HS	56.3 ± 3.35 d	4.03 ± 0.09 d	1.09 ± 0.07 a	1.34 ± 0.05 d	1.44 ± 0.05 d	0.65 ± 0.03 c
*erectus*	MS + SA	71.3 ± 2.66 ab	5.52 ± 0.24 b	0.72 ± 0.01 c	1.87 ± 0.06 b	1.99 ± 0.05 b	0.83 ± 0.03 a
	HS + SA	68.0 ± 2.61 bc	4.35 ± 0.35 c	0.95 ± 0.08 a	1.65 ± 0.06 c	1.71 ± 0.06 c	0.85 ± 0.02 a
	C	64.0 ± 1.70 c	4.52 ± 0.17 c	0.61 ± 0.01 d	1.55 ± 0.20 c	1.41 ± 0.15 d	0.89 ± 0.04 a
	MS	48.7 ± 1.52 de	3.64 ± 0.21 de	0.75 ± 0.09 c	1.14 ± 0.05 e	1.09 ± 0.07 e	0.76 ± 0.01 b
*Populus*	HS	32.0 ± 1.47 f	3.02 ± 0.14 e	0.81 ± 0.01 b	0.86 ± 0.07 f	0.84 ± 0.01 f	0.66 ± 0.02 c
*deltoides*	MS + SA	53.3 ± 1.52 d	3.91 ± 0.17 d	0.82 ± 0.08 b	1.34 ± 0.06 d	1.29 ± 0.06 d	0.99 ± 0.06 a
	HS + SA	43.1 ± 1.87 e	3.51 ± 0.12 d	0.58 ± 0.10 c	1.21 ± 0.14 e	1.21 ± 0.18 d	0.84 ± 0.04 a
S-effect		*p* < 0.001	*p* = 0.021	*p* = 0.161	*p* < 0.001	*p* < 0.001	*p* < 0.001
T-effect		*p* < 0.001	*p* = 0.051	*p* < 0.001	*p* < 0.001	*p* < 0.001	*p* < 0.001
S × T effect		*p* < 0.001	*p* = 0.405	*p* = 0.005	*p* = 0.046	*p* = 0.046	*p* < 0.001

**Table 2:** Effect of water deficit and SA application on osmolyte accumulation of *Conocarpus erectus* and *Populus deltoides*. Each value represents the mean (± SE) of species in different treatment combinations. Small letters represent significant differences among the species and treatments tested using the post-hoc Turkey’s HSD test. All tests were considered significant at *p* < 0.05.

Species	Treatments	Pc (µmol g^−^^1^ FW)	TSS (mg g^−^^1^ FW)	TPC (mg g^−^^1^ FW)	SP (mg g^−^^1^ FW)
	C	20.1 ± 0.53 d	74.3 ± 0.63 c	1.38 ± 0.02 ef	23.6 ± 0.49 d
	MS	24.7 ± 0.94 c	83.0 ± 1.12 b	2.20 ± 0.05 c	28.6 ± 0.30 b
*Conocarpus*	HS	29.0 ± 0.39 b	84.3 ± 1.00 b	2.55 ± 0.02 b	33.2 ± 0.63 a
*erectus*	MS + SA	30.2 ± 0.48 b	86.9 ± 1.17 a	2.54 ± 0.02 b	31.6 ± 0.45 a
	HS + SA	34.9 ± 0.58 a	88.6 ± 1.25 a	2.82 ± 0.02 a	33.6 ± 0.90 a
	C	19.0 ± 0.38 e	69.3 ± 1.39 e	1.25 ± 0.01 f	24.8 ± 0.32 d
	MS	22.4 ± 1.12 cd	74.6 ± 0.46 d	1.59 ± 0.04 e	26.9 ± 1.12 c
*Populus*	HS	26.5 ± 0.42 c	83.8 ± 0.93 b	2.1 ± 0.03 cd	30.0 ± 0.71 ab
*deltoides*	MS + SA	29.6 ± 0.63 b	78.5 ± 0.87 c	1.83 ± 0.01 d	28.7 ± 0.86 b
	HS + SA	30.1 ± 0.32 b	89.3 ± 1.63 a	2.35 ± 0.04 c	32.3 ± 0.89 a
S-effect		*p* < 0.001	*p* < 0.001	*p* < 0.001	*p* < 0.001
T-effect		*p* < 0.001	*p* < 0.001	*p* < 0.001	*p* < 0.001
S × T effect		*p* < 0.001	*p* < 0.001	*p* < 0.001	*p* < 0.001

## Data Availability

Data is available on request from the corresponding author.
